# Data-driven formulation of steel fiber pull-out force in cementitious composites using genetic programming

**DOI:** 10.1038/s41598-025-22595-x

**Published:** 2025-11-04

**Authors:** Ali Kooshkaki, Seyed Ali Emamian, Ramin Kazemi, Amir H. Gandomi

**Affiliations:** 1 Department of Civil Engineering, Hakim Sabzevari University, Sabzevar, Iran; 2 Department of Civil and Water Engineering, Laval University, Québec, Canada; 3 Independent Researcher, Sabzevar, Iran; 4 Faculty of Engineering & Information Technology, University of Technology Sydney, Ultimo, 2007 Australia; 5 University Research and Innovation Center (EKIK), Obuda University, Budapest, 1034 Hungary; 6 Department of Computer Science, Khazar University, Baku, Azerbaijan

**Keywords:** Pull-out force, Steel fiber, Cementitious composites, Gene expression programming, Sensitivity analysis, Engineering, Materials science

## Abstract

This study aims to develop a data-driven approach for predicting and formulating the pull-out force of steel fibers in cementitious composites using a genetic programming variant, gene expression programming (GEP). A comprehensive dataset of 437 experimental data was collected from previous studies, including key variables such as embedment length, fiber inclination angle, tensile strength of fibers, aspect ratio, loading rate, water-to-cement ratio, compressive strength of matrix, and fiber geometry. The GEP model developed in this study demonstrated notable accuracy in predicting pull-out force, with an R^2^ of 0.93. Model performance was evaluated using multiple statistical criteria, confirming its satisfactory predictive ability. Furthermore, a k-fold cross-validation was performed, and the results confirmed the model’s robustness and capability for generalizing to new data. Sensitivity analysis using SHAP interpretation revealed that the fibers’ tensile strength and the embedment length are the most influential factors affecting the pull-out force. The GEP method was adopted for its ability to generate accurate and interpretable mathematical formulas. Accordingly, a mathematical formula for the pull-out force is proposed, providing an efficient and interpretable tool for future studies. Overall, the GEP approach can significantly reduce reliance on costly and time-consuming experimental procedures while ensuring reliable performance predictions and practical formulations.

**Supplementary Information:**

The online version contains supplementary material available at 10.1038/s41598-025-22595-x.

## Introduction

### Background and literature review

**Fig. 1:**
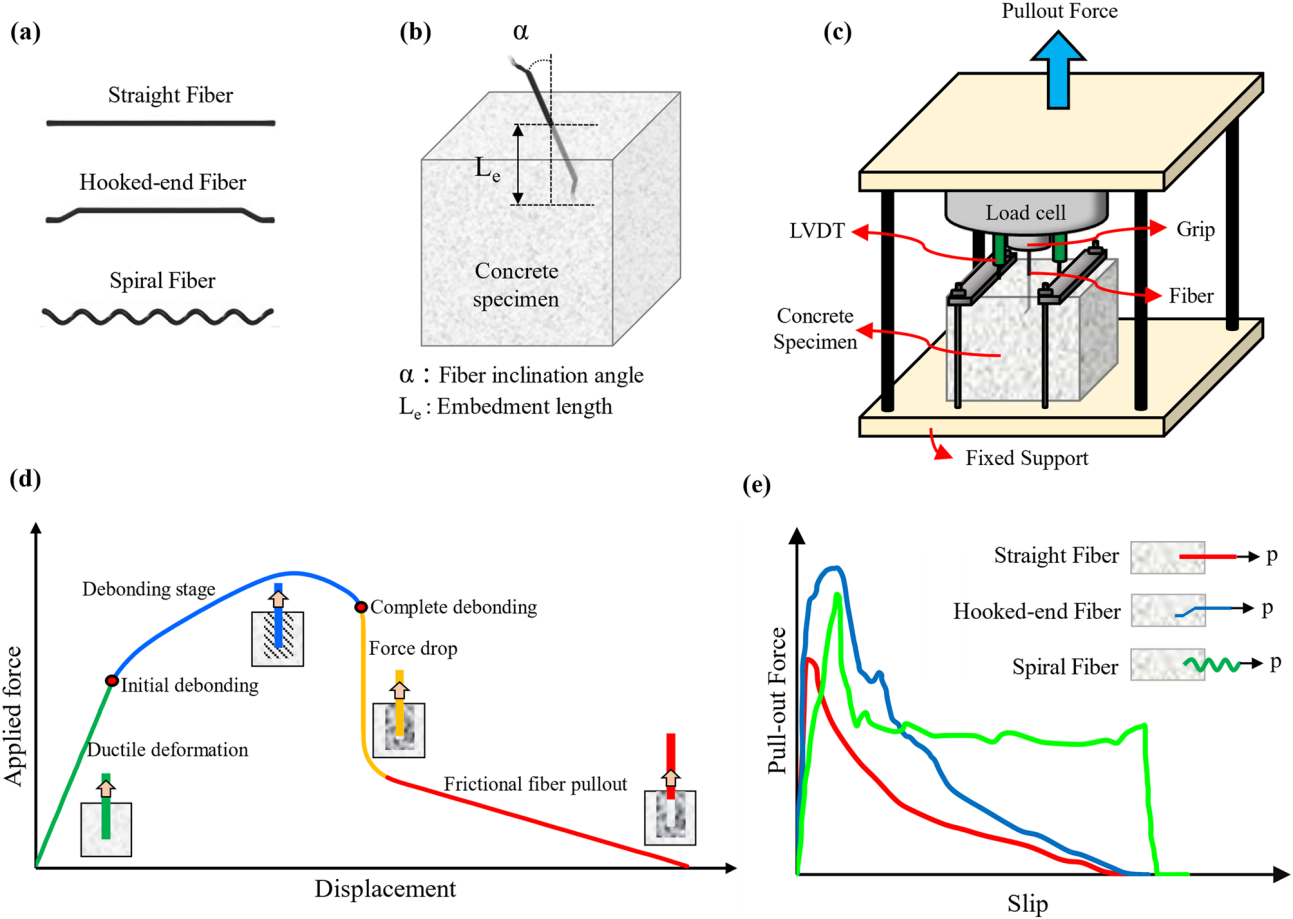
An in-depth overview of the mechanism of fiber pull-out test: (a) fibers profile, (b) pull-out specimen, (c) pull-out test setup, (d) an idealized force-slip diagram, and (e) force-slip behavior of three types of fibers.

Cementitious composites are quasi-brittle materials, with key properties of concern include tensile strength, toughness, and crack resistance^1–3^. Therefore, various efforts, such as reinforcing cementitious composites with different types of fibers and rebars, have been conducted to enhance cementitious composites’ brittleness and crack resistance^4–6^. The use of various fibers is one of the common approaches for improving the ductility of cementitious composites. Fibers can effectively bridge the micro-cracks, enhance the cementitious composites’ ability to resist higher tensile stresses, and improve the flexibility, resilience, and durability of cementitious composites^7,8^. Different types of fibers, such as natural^9^, synthetic^10^, glass^11^, steel^12^, etc., have been used in previous research. However, steel fibers with different geometries, such as straight, hooked-end, and spiral, as shown in Fig. 1a, are most commonly used due to their higher tensile strength, which enhance the tensile strength of cementitious composites, and they are also used in high-strength concrete^13,14^. Moreover, steel fibers have greater energy absorption and enhanced durability^15,16^.

A key property of fiber-reinforced cementitious composites is the bond between the matrix and the fibers^17,18^. This behavior has been studied under various influencing factors, such as loading rate and fiber inclination angle, using the single fiber pull-out test^19,20^. The pull-out specimen and test setup are illustrated in Figs. 1b and c, respectively. Additionally, an idealized force-slip diagram of a single fiber pull-out test is shown in Fig. 1d, representing the four test stages. The first stage is marked by ductile deformation, indicating that the specimen (containing both fiber and matrix) behaves in a ductile manner. During this stage, no debonding occurs between the fiber and the cementitious matrix, and the interface remains intact. As this stage concludes, interface debonding begins, leading to the formation of cracks in the cementitious matrix. These cracks propagate, and debonding continues until the entire embedded length of the fiber is debonded, resulting in a noticeable drop in force, as depicted in the diagram. Ultimately, once the fiber is fully debonded, the only resistance to fiber pull-out is the friction between the fiber and the matrix, which continues until the fiber is fully extracted from the specimen. However, as indicated in Fig. 1e, the Fp-slip diagram may vary slightly due to differences in fiber geometry. Many studies have investigated influential factors on the pull-out behavior of steel fibers. Wang et al.^20^ investigated the effect of corrosion and inclination on the pull-out behavior of different steel fibers. The results indicated that bond strength decreases by increasing the inclination angle from 0º to 45º for uncorroded samples with crimped and milled-cut steel fibers. On the contrary, the bond strength of the uncorroded samples with hooked-end fibers increases by increasing the inclination angle. Also, it was shown that corrosion decreases the bond strength in samples with crimped and milled-cut fibers, while for hooked-end samples, the bond strength increases after corrosion. Peyvandi et al.^21^ examined the pull-out behavior of spiral steel fibers. Their results revealed that spiral fibers offer a convincing energy absorption capacity. Also, it was shown that increasing the inclination angle from 0º to 60º leads to a maximum 50% decrease in pull-out capacity.

Recently, artificial intelligence (AI) methods have been increasingly utilized to predict various properties of cementitious materials^22,23^. These approaches aim to enhance efficiency and accuracy, reduce costs, and optimize laboratory resource usage. Table 1 summarizes the most recent research on using different AI methods to predict the properties of steel fiber-reinforced cementitious composites (SFRCC). Among the AI methods, such as machine learning (ML)^24–29^, artificial neural networks (ANN)^30–33^ and other data-driven methods^34–38^, GEP is an effective prediction tool, excelling at modeling complex, non-linear relationships while producing interpretable mathematical expressions that offer accuracy and transparency^39,40^, and it has successfully been used in various studies to predict different properties of cementitious materials^41–43^.

**Table 1:** Some recent research on using different AI methods for predicting SFRCC properties.

Authors [Ref.]	Prediction target	Type of fiber used	Best proposed method
Zhang et al.^44^	Compressive strength Splitting tensile strength	Steel fiber	GBRT
Que et al.^45^	Tensile strength	Smooth straight steel fiber	GB
Mehedi et al.^46^	Slump value	Steel fiber	RF
	Compacting factor		
	Compressive strength		
	Splitting tensile strength		
Ali et al.^47^	Compressive strength	Hooked-end steel fiber	GEP
Pakzad et al.^48^	Splitting tensile strength	Hooked-end industrial steel fiber	SVR
Arif et al.^49^	Compressive strength	Steel fiber	GEP

GBRT: Gradient boosted regression tree, GB: Gradient boosting, RF: Random forest, GEP: Genetic expression programming, SVR: Support vector regression.

In parallel, several recent studies have employed advanced AI techniques, such as ensemble learning and hybrid models, to predict the mechanical performance of concrete composites incorporating various materials and admixtures^50–53^, and to evaluate their behavior under different loading conditions^54,55^. These developments highlight the growing role of AI in modeling complex concrete behavior. Unlike traditional ML models that function as black boxes^56^, GEP provides interpretable mathematical expressions, enhancing both predictive accuracy and practical usability in engineering applications.

So far, only a few limited studies have attempted to analytically predict the pull-out behavior of steel fibers from cementitious materials^57–59^. Hemmatian et al.^60^ predicted the maximum pull-out force (Fp) and the corresponding bond slip using the ANN method on 382 collected experimental data, and the best prediction model reached an overall coefficient of determination (R^2^) of 0.91. Similarly, Huang et al.^61^ proposed an ML-based prediction equation for Fp in UHPC based on an XGBoost model and experimental data. While their approach included fiber shape correction factors, the final equation reached a modest level of accuracy, with an R² of 0.74. However, using other AI techniques, providing a mathematical formulation, and utilizing a more comprehensive and newer dataset is suggested in the mentioned studies.

### Research significance

**Fig. 2:**
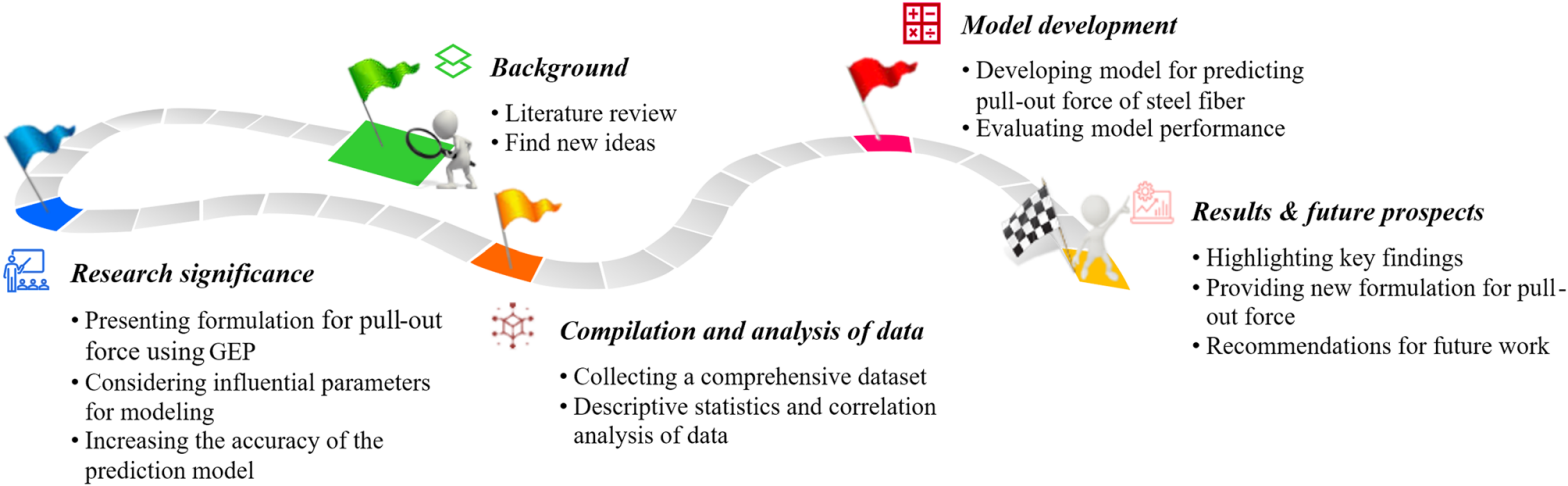
The roadmap of the current research.

Many experimental studies have used the single-fiber pull-out test to assess the interaction and bonding properties between the fibers and the cementitious composites. However, laboratory programs consume much energy and time and waste considerable resources. On the other hand, utilizing AI methods can effectively minimize challenges from experimental approaches. However, there is a lack of AI modeling studies that predict the pull-out behavior of steel fibers from cementitious composites. Hemmatian et al.^60^, in recommendations for future works, suggested using other AI methods to enhance prediction accuracy and provide a mathematical formula for calculating the fiber Fp of cementitious composites. Therefore, this study aims to predict the maximum Fp using the GEP method and present a formula for the Fp of steel fibers in SFRCC. Besides, a more comprehensive dataset, including 437 data points for Fp, is used to prepare the prediction model, making it more reliable than previous research. For a concise overview, the roadmap of the current study is illustrated in Fig. 2.

## Research methodology

This section describes the methodology utilized to create a predictive model for the Fp of SFRCC. Gene expression programming (GEP) is used to formulate experimental approaches, taking advantage of its capacity to capture complex nonlinear relationships between input variables and output responses. This method guarantees the creation of precise, data-driven predictions for essential mechanical properties by uncovering detailed correlations within the dataset.

### Overview of GEP

GEP is an advanced extension of genetic algorithms (GA), introduced by Holland^62^, and genetic programming (GP), proposed by Koza^63^. Like its predecessors, GEP is based on three main steps: initializing a population, evaluating fitness, and genetic operations like crossover and mutation. Unlike GA, where individuals are represented as fixed-length linear chromosomes, and GP, which uses nonlinear parse trees, GEP utilizes a hybrid approach. It encodes individuals as chromosomes, which are expressed as nonlinear structures called expression trees (ETs)^64^.

The main distinction between GA, GP, and GEP lies in the structure of their individuals. In GA, individuals are represented as fixed-length linear chromosomes. GP uses nonlinear parse trees of varying sizes and shapes^65^. In contrast, GEP combines these approaches by representing individuals as chromosomes that are expressed as nonlinear entities, called expression trees^64^.

**Fig. 3:**
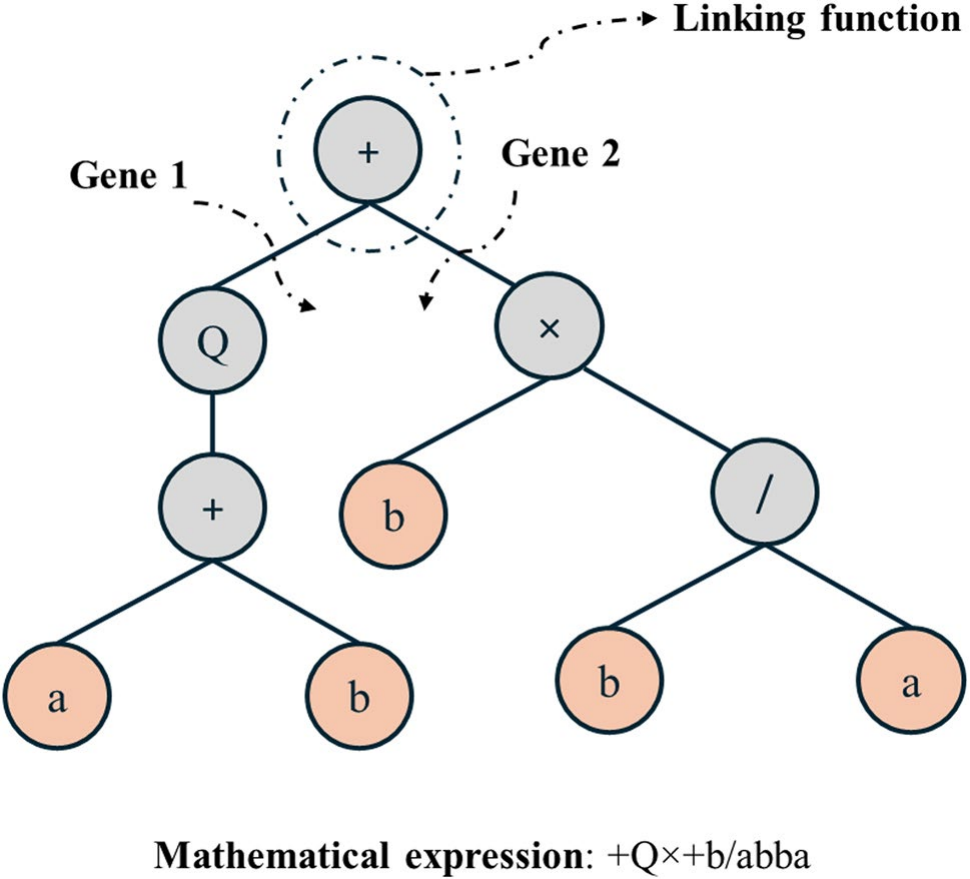
Illustration of chromosome decoding in GEP^43^.

In GEP, problems are represented through expression trees composed of operators, functions, constants, and variables. For example, as shown in Fig. 3, mathematical formulations such as $\sqrt {(a+b)} +(b \times (\frac{b}{a}))$ can be represented as an expression tree, clearly showing its algebraic structure^64^. This feature makes GEP highly adaptable for solving complex problems.

GEP also addresses some limitations of GP. While GP uses nonlinear structures that act as both the genotype and phenotype, GEP separates these roles, transmitting only the genome (genotype) to subsequent generations^65^. This distinction allows GEP to generate simpler and more interpretable solutions compared to GP, which often produces overly complex expressions. Additionally, GEP introduces a significant feature: chromosomes are composed of multiple genes of fixed length, which include terminal constants and arithmetic functions^43^. A unique decoding mechanism, referred to as Karva notation, is employed in GEP to interpret the information stored within chromosomes, facilitating the development of empirical models^65^. This innovation allows GEP to efficiently represent and solve problems while maintaining its adaptability and accuracy.

### Compilation and analysis of database

In this study, we compiled an comprehensive database from previously published literature^21,66–76^, comprising a dataset of 437 mixtures to predict the Fp of SFRCC. To ensure high data quality, a thorough preprocessing was conducted: only samples with complete input and output parameter records were retained. Studies missing essential metadata or using unconventional testing procedures were excluded. Additionally, duplicates were identified and removed by cross-referencing sample IDs and test conditions, and any samples missing key parameters were excluded. Table 2 provides an overview of the data sources, number of samples, and types of fibers used, specifically, three types of steel fibers: straight, spiral-deformed, and hooked-end. Detailed dataset information is available in the supplementary file.

**Table 2:** Summary of data sources.

No. ID	Authors [Ref.]	Year	Number of samples	Fiber type
1	Abu-Lebdeh et al.^66^	2010	29	Straight and hook- end steel fiber
2	Abu-Lebdeh et al.^67^	2011	10	Straight and hook- end steel fiber
3	Tuyan and Yazıcı^68^	2012	8	Hook-end steel fiber
4	Zeighami et al.^69^	2016	40	Hooked-end steel fibers
5	Tai and El-Tawil^70^	2017	17	Straight and hook- end steel fiber
6	Abdallah et al.^71^	2017	6	Hooked-end steel fibers
7	Lin and Ostertag^72^	2017	79	Hooked-end steel fibers
8	Deng et al.^73^	2018	31	Straight and hook- end steel fiber
9	Yoo et al.^74^	2019	8	Straight and hook- end steel fiber
10	Yoo and Kim^75^	2019	16	Straight and hook- end steel fiber
11	Peyvandi et al.^21^	2022	166	Spiral deformed steel fiber
12	Kim and Dang^76^	2023	27	Straight steel fiber

**Fig. 4:**
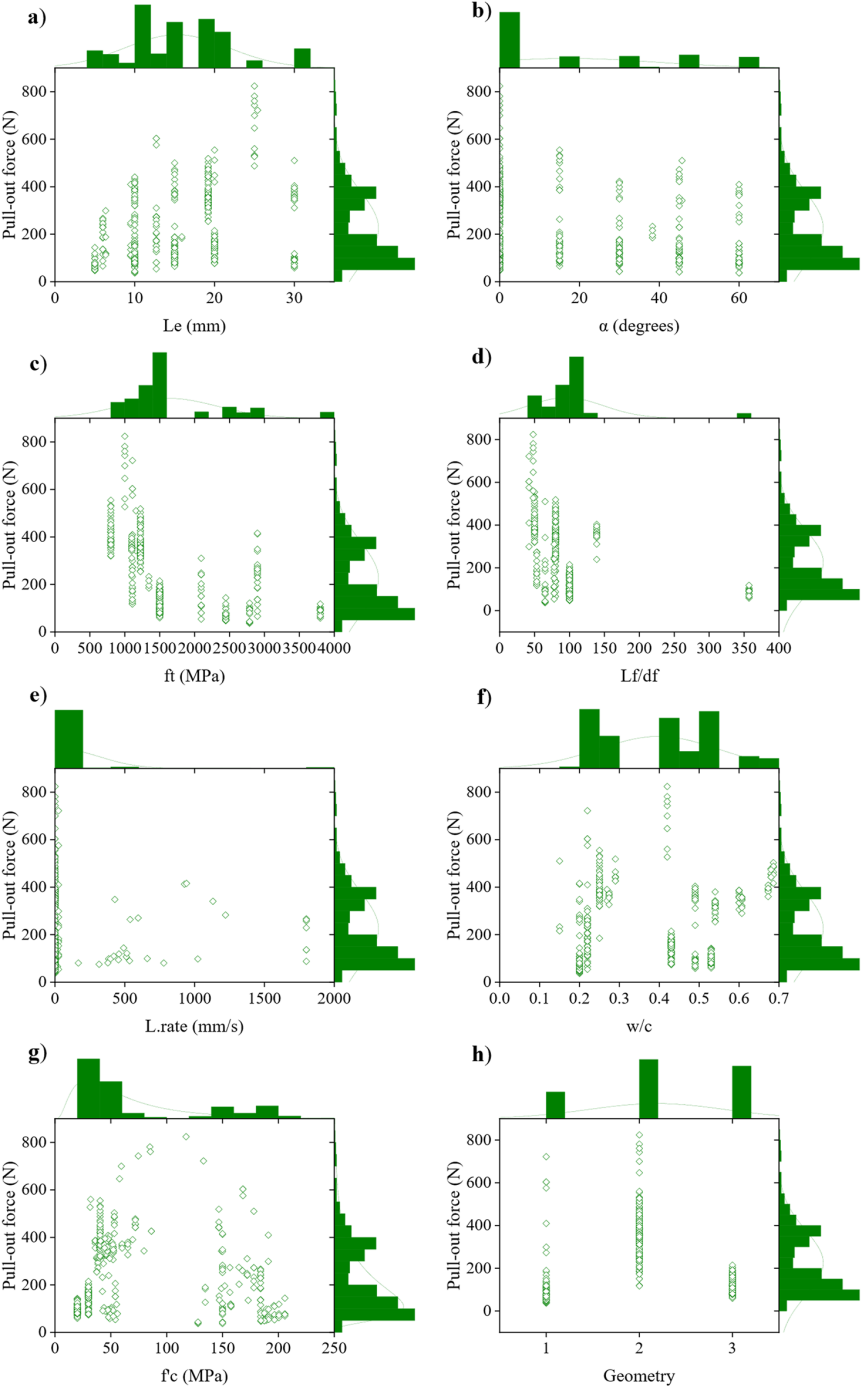
Scatter plots with marginal histograms illustrating the relationship between Fp and each input variable in SFRCC.

Eight critical input variables were selected to develop a reliable predictive model. These variables included embedded length (L_e_), inclination angle (α), fiber tensile strength (ft), fiber aspect ratio (Lf/df), loading rate (L.rate), water-to-cement ratio (w/c), compressive strength of matrix (f’c), and fiber geometry categorized as 1 straight steel fibers, 2 hooked-end steel fibers, and 3 spirally deformed steel fibers. Analyzing the distribution of these input variables is essential for assessing the model’s generalizability. The histograms in Fig. 4 illustrate the frequency distribution of each input and output variable across various mix designs, along with the range and prevalence of these variables. This frequency analysis reveals the distribution and effect of each variable on the Fp of SFRCC. For instance, L_e_ exhibits a moderate positive trend with Fp, with values ranging between 5 and 30 mm. The inclination α ranges from 0° to 60°, with a distribution skewed towards lower values, indicating a higher frequency of small angles. The ft ranges between 800 and 3800 MPa, and most values are below 1500 MPa, revealing that a majority of the fibers are lower-strength fibers. The Lf/df is mainly distributed below 120, indicating that higher aspect ratios are less frequent. The L.rate shows a wide range of variation, from 0.008 to 1800 mm/s. However, the majority of the values fall below 600 mm/s, suggesting that the loading rates are generally moderate. The w/c ranges from 0.15 to 0.68, with most values distributed around 0.2–0.5. The f’c varies from 20 to 205.8 MPa, with most values below 100 MPa. Lastly, geometry illustrates different types of steel fibers, which are categorized into three separate groups.

The details of some statistical parameters, such as average, standard deviation, skewness, and kurtosis, which demonstrate the variable’s impact on the predictive model, can be found in Table 3. For example, the average and standard deviation of ft and L.rate are (1624.81, 695.52), and (60.41, 265.30), respectively, indicating central tendency and variability. Also, the skewness and kurtosis of Lf/df and L.rate are (3.93, 16.90) and (5.33, 29.73), respectively, revealing asymmetry and peaked distributions. On the other hand, positive skewness values (e.g., fiber tensile strength, loading rate) indicate right-tailed data, while negative values (e.g., geometry of fiber) suggest left-tailed distributions. High kurtosis values (e.g., fiber aspect ratio, loading rate) point to heavy-tailed and peaked distributions, whereas negative values (e.g., water-to-cement ratio) reflect flatter and lighter-tailed distributions. These statistical insights are critical for assessing data quality and guiding model development, particularly in accurately capturing the behavior of SFRCC under varied conditions.

**Table 3:** Descriptive statistics of input and output variables for sfrcc’s pull-out model.

Variable	Symbol	Unit	Category	Statistics				
				Range	Average	Standard deviation	Skewness	Kurtosis
Embedment length	L_e_	mm	Input	5–30	15.29	6.50	0.47	-0.21
Fiber inclination	α	Degrees	Input	0–60	17.34	21.73	0.82	-0.86
Fiber tensile strength	ft	MPa	Input	800–3800	1624.81	695.62	1.45	1.61
Fiber aspect ratio	Lf/df	-	Input	42-357.14	94.88	53.66	3.93	16.90
Loading rate	L.rate	mm/s	Input	0.008–1800	60.41	265.30	5.33	29.73
Water to cement ratio	w/c	-	Input	0.15–0.686	0.40	0.15	-0.04	-1.34
Compressive strength of matrix	f’c	MPa	Input	20-205.8	69.93	60.16	1.12	-0.43
Geometry of fiber	Geometry	-	Input	1–3	2.19	0.73	-0.31	-1.10
Pull-out force	Fp	N	Output	37.19–824	224.64	152.30	0.99	0.63

**Fig. 5:**
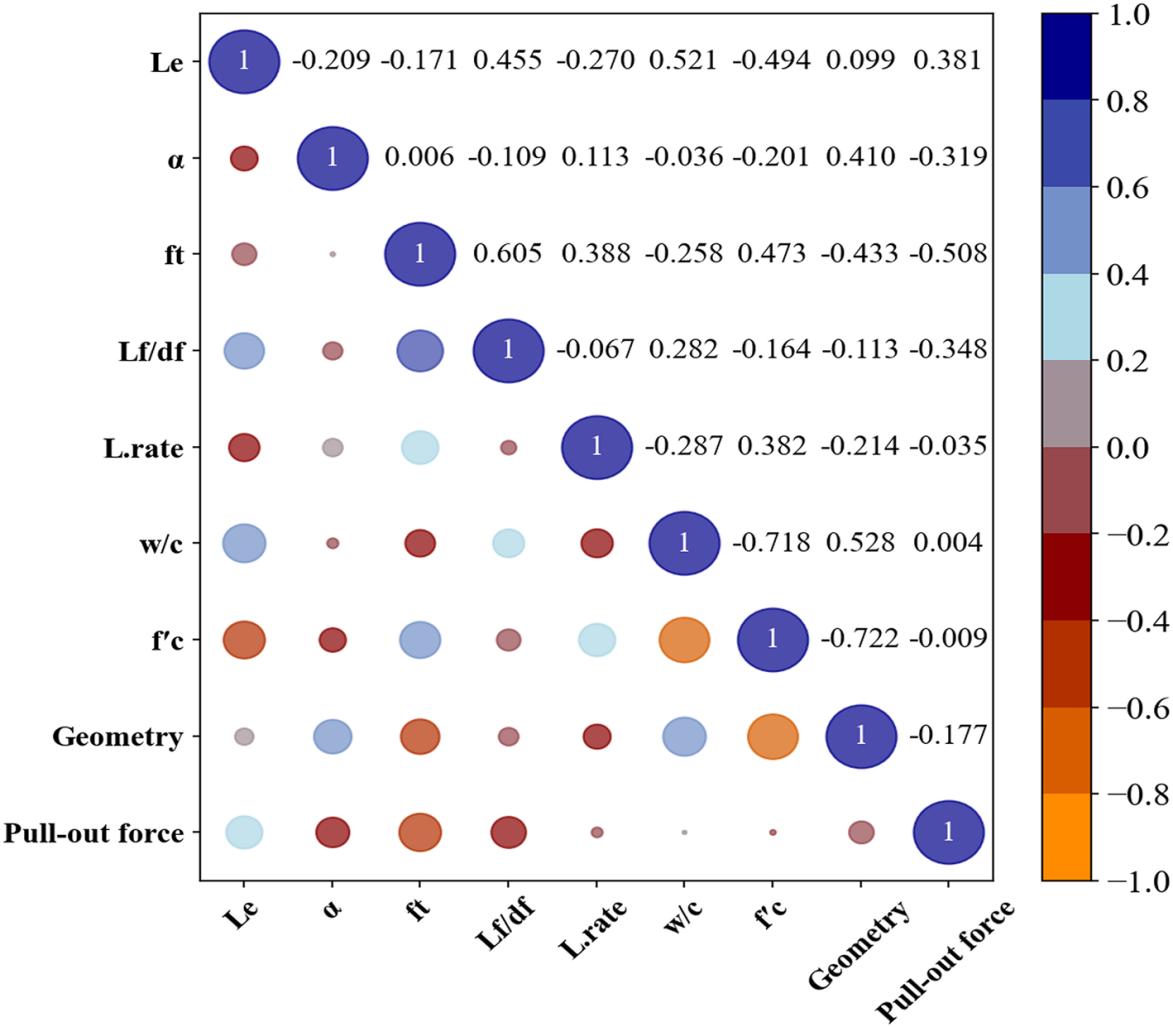
Pearson correlation coefficients for fiber Fp in SFRCC.

Furthermore, the Pearson correlation coefficients (PCCs) depicted in Fig. 5 highlight the linear relationships between input and output variables in predicting the Fp of SFRCC. In general, PCCs range from + 1 to -1. Values close to + 1/ -1 indicate a strong positive/negative correlation, while the values around 0 indicate a weak or no linear relationship. Figure 5 reveals that there is a moderate negative correlation between ft and Fp of -0.508, illustrating that as ft increases, Fp tends to decrease slightly, possibly due to changes in bond characteristics between the fiber and matrix^77^. As a result, considering ft as a factor affecting Fp performance is unavoidable. By identifying key relationships, the model can capture the behavior of SFRCC more accurately under varied conditions, enhancing its predictive value for Fp^78^. The importance of these correlation assessments in enhancing the predictive model is undeniable.

### Model development

In this research, we employ GEP, a variant of GP, to predict the Fp of SFRCC using the GeneXpro Tools software (Version 5.0)^79^. The development of the GEP models involves five major steps^80^. The first step is selecting the fitness function, which evaluates the quality of individuals in the population, as shown in Eq. (1).

1 $$f_{i} = \sum\limits_{{j = 1}}^{{C_{t} }} {\left[ {M - {\text{ }}\left| {C_{{ij}} - T_{j} } \right|} \right]}$$

Where M represents the variety of selection, C_ij_ is the returned value for the *i*-th individual for the *j*-th fitness case, C_t_ is the total number of fitness cases, and T_j_ is the target value for the *j*-th fitness case. The term $\left| {C_{{ij}} - T_{j} } \right|$ represents prediction error; and if the error ≤ 0.01, the fitness function reaches its maximum value ${f_i}={f_{\hbox{max} }}={C_t} \times M$^81^. This mechanism enables the system to iteratively optimize itself to find the best solution^81^.

In the second step, a set of terminals (T) and functions (F) is defined. The F set includes basic mathematical operators, including subtraction [-], addition [+], multiplication [×], division [÷], and other advanced mathematical functions (e.g. Sqrt, 3Rt, x^2^, x^3^, x^4^, x^5^, Neg).

The third step involves defining the chromosomal architecture, including determining the head length and the number of genes in each chromosome. Various configurations are tested, and the optimal chromosome structure is selected based on performance.

In the fourth step, linking functions, such as addition, subtraction, multiplication, or division, are chosen to combine sub-ETs. This study used the additional linking function to link the sub-ETs.

**Fig. 6:**
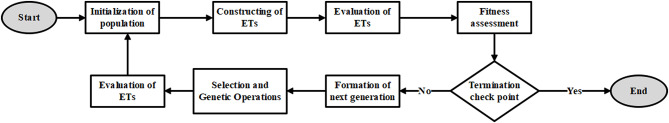
GEP algorithm flowchart^80^.

Finally, the fifth step is selecting the genetic operators. A combination of genetic operators, including crossover and mutation, is applied to evolve the population and improve the model’s performance^81^. A visual representation of the GEP algorithm is provided in Fig. 6. These systematic steps allow GEP to efficiently develop accurate models by leveraging a combination of genetic programming principles and advanced mathematical formulations.

### Evaluation criteria

The performance of the prediction model is evaluated using six statistical metrics. Detailed explanations of each metric are provided below:

- ***Root mean squared error (RMSE)***: A recognized metric for evaluating the machine learning model’s performance. It measures the average difference between predicted and actual values, and lower RMSE values indicate more accurate predictions. Due to its versatility across various domains, RMSE simplifies comparing the performance of different models. Additionally, it is straightforward to understand and provides a clear interpretation of model accuracy. It can be calculated using Eq. (2).

2 $$RMSE=\sqrt {\frac{1}{n}\sum\limits_{{i=1}}^{n} {({A_i}} - {P_i}{)^2}}$$

- ***Mean absolute error (MAE)***: Another popular metric for measuring the machine learning model’s performance. It measures the average absolute difference between predicted and actual values. MAE values can range from 0 to ∞, while a lower MAE indicates better model performance. It is easy to understand and less sensitive to outliers compared to RMSE because it does not square the differences. The MAE values can be measured as follows:

3 $$MAE=\frac{{\sum\limits_{{i=1}}^{n} {\left| {{A_i} - {P_i}} \right|} }}{n}$$

- ***Coefficient of determination (R***^***2***^***)***: It is a statistical metric that shows the effectiveness of a regression model in fitting the data. It indicates the fraction of the total variance in the actual values that is explained by the predicted values. The R^2^ value range is 0 to 1, with higher values reflecting better accuracy. R^2^ values can be used to evaluate the effectiveness of a model. R^2^ values can be derived using the following formula:

4 $${R^2}={\left( {\frac{{\sum\limits_{{i=1}}^{n} {({A_i} - \bar {A})} ({P_i} - \bar {P})}}{{\sqrt {\left[ {\sum\limits_{{i=1}}^{n} {{{({A_i} - \bar {A})}^2}} } \right]\left[ {\sum\limits_{{i=1}}^{n} {{{({P_i} - \bar {P})}^2}} } \right]} }}} \right)^2}$$

- ***Variance accounted for (VAF)***: It is a statistical measure that shows how much of the total variability in a dataset is explained by a specific model. It assesses the model’s fit and predictive power, and ability to describe underlying patterns. The VAF typically ranges is 0 to 100. High VAFs indicate that the model effectively explains the data, while low VAFs indicate refinement needs or other influencing factors. This measure is crucial for evaluating a model’s reliability for prediction and analysis. VAF can be calculated using the following formula:

5 $$VAF\% =\left[ {1 - \frac{{\operatorname{var} (A - P)}}{{\operatorname{var} (A)}}} \right] \times 100$$

- ***Nash–Sutcliffe efficiency (NSE)***: It is a commonly employed statistical measure for assessing the performance of machine learning models. The NSE value allows for a comparison between predicted values and experimental values. The NSE ranges from -∞ to 1. A low or negative NSE value implies poor model calibration, whereas a higher NSE value indicates strong performance. Therefore, NSE is critical for evaluating and improving the quality of machine learning models. The NSE value can be computed as follows:

6 $$NSE=1 - \frac{{\sum\limits_{{i=1}}^{n} {{{({A_i} - {P_i})}^2}} }}{{\sum\limits_{{i=1}}^{n} {{{({A_i} - \bar {A})}^2}} }}$$

- ***a20-index***: The value of this metric lies in its easy-to-understand interpretation as the percentage of predictions within an acceptable range, its emphasis on a practical ± 20% error margin, and its capability to determine if a model meets reliability standards. The A-20 index ranges from 0 to 100, with values closer to 100 indicating superior model performance. The formula for the a-20 index is as follows:

7 $$a - 20\,index=\frac{{{m_{20}}}}{n} \times 100$$

where;

$$\begin{gathered} {A_i}:{\text{ denotes the actual values}} \hfill \\ {P_i}:{\text{denotes the predicted values of }}{A_i} \hfill \\ \overline {A} :{\text{denotes the avearge of actual values }} \hfill \\ \overline {P} :{\text{denotes the avearge of predicted values}} \hfill \\ n{\text{ :}}{\text{ denote the total number of data point}} \hfill \\ {m_{20}}:{\text{denotes the number of samples with actual value/predicted value ratio between 0}}{\text{.8 and 1}}{\text{.2}} \hfill \\ \end{gathered}$$

Evaluation metrics are derived from all predicted and actual values in the dataset (i.e., the sum of values), rather than from individual data points. As a result, values near zero do not impact the outcome, and the metric remains valid even when some values are zero.

### Cross-validation

**Fig. 7:**
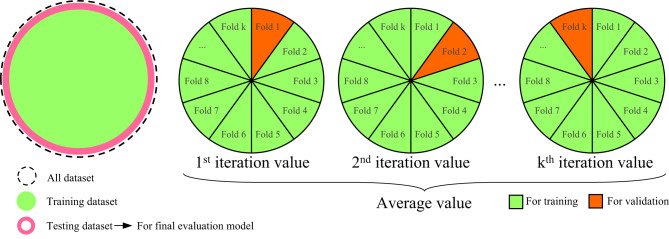
K-fold cross-validation process.

K-fold cross-validation is an effective method in machine learning for assessing the performance of a model and its capability for generalizing to new data^82–84^. The procedure starts with dividing all data into two training and testing groups, while the training dataset is used in the first k-fold process. This procedure splits the mentioned dataset into k equal-sized groups, known as “folds.” The model experiences training for k times, with each fold serving as the validation set once, while the remaining k-1 folds are used for the training set. This procedure continues until every fold has been utilized once as the validation set. The results from all iterations are then averaged to obtain a complete performance measurement. Finally, for the final model evaluation, the obtained average values undergo evaluation with the testing dataset. This strategy reduces the chances of overfitting and offers a more reliable estimate of the model’s performance by using the entire dataset for training and validation without any overlap. A visual representation of this procedure is demonstrated in Fig. 7.

### SHAP analysis

SHAP (Shapley Additive exPlanations) is a reliable framework commonly used to interpret AI models by assessing how much each input variable contributes to the model’s predictions^85^. SHAP offers a consistent method for explaining individual predictions by linking the model output to the input variable. It determines the average marginal contribution of a feature across all possible feature combinations to clarify how a model makes its predictions. SHAP values can be visualized to show important variable and their impacts on predictions. Also, this tool is essential for reducing the ambiguities in complex models, enhancing reliability, and enabling transparency in AI systems.

## Results and discussion

### Performance of the developed model

**Fig. 8:**
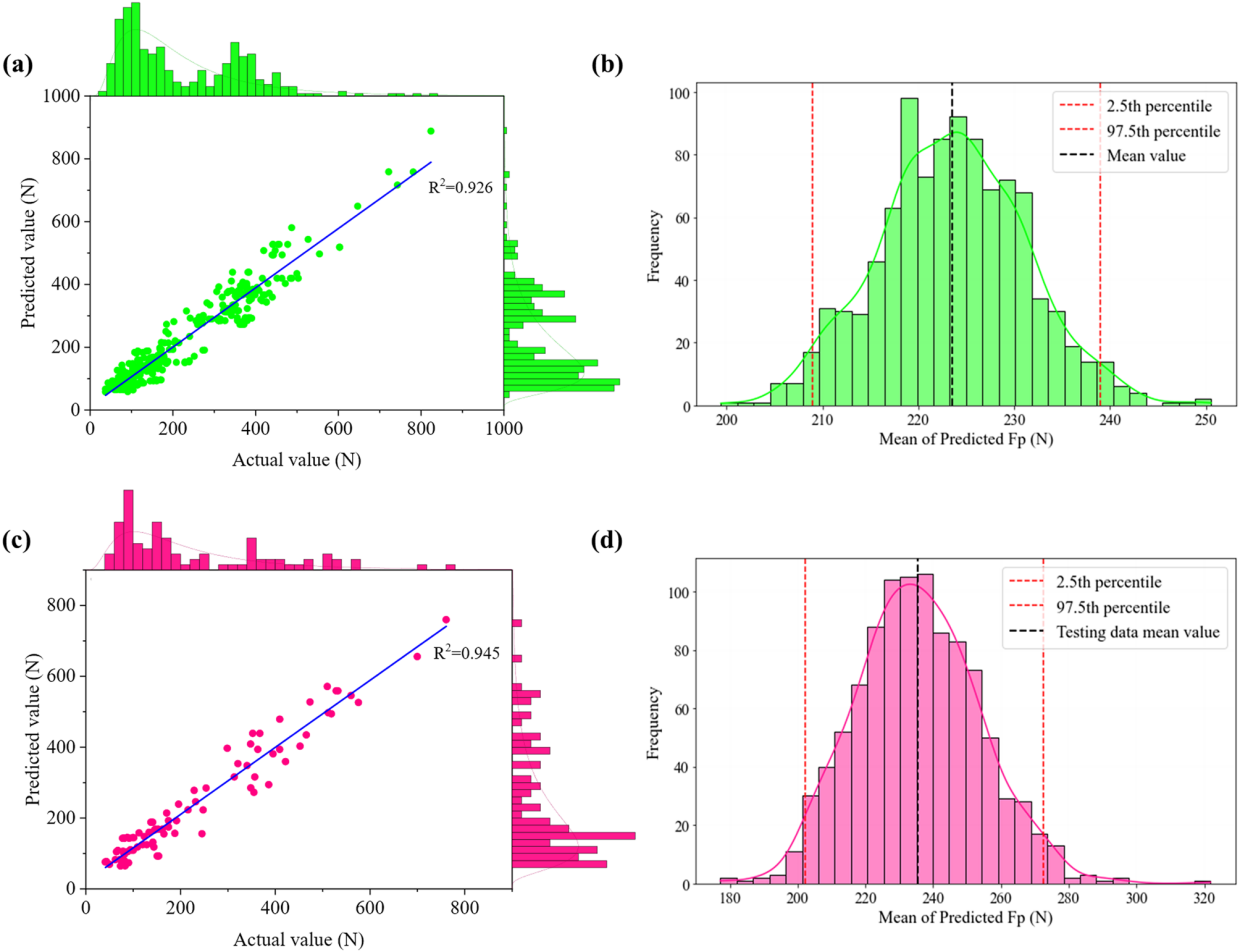
Predicted vs. actual values with marginal distribution histograms and bootstrap distributions of the predicted Fp: (a) and (b) for the training dataset, and (c) and (d) for the testing dataset.

**Fig. 9:**
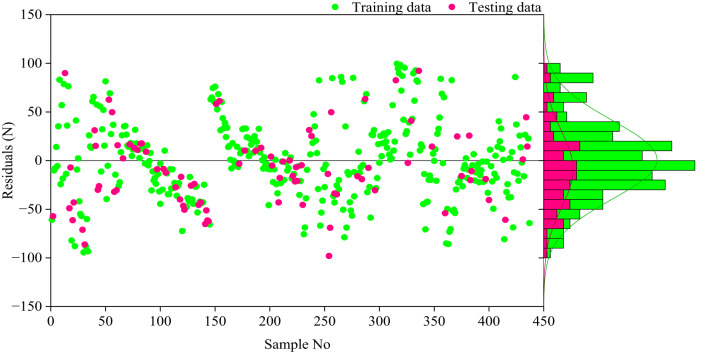
Residual values and their frequency histogram.

**Fig. 10:**
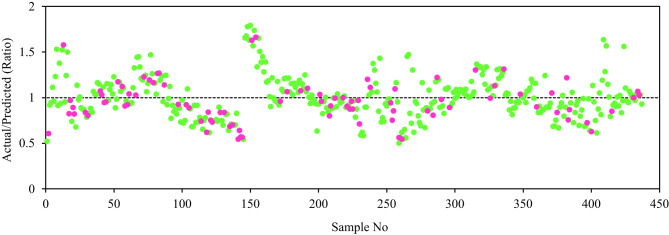
Actual/predicted ratio of Fp values.

The genetic parameters used to develop the proposed GEP model are summarized in Table 4. It is worth noting that the genome parameters were determined through a combination of empirical tuning, dataset-driven adjustments, and a previously published study^56^. These carefully optimized parameters were selected to ensure effective modeling of the Fp in SFRCC. In the GEP model, key features include chromosome number, head size, gene number, linking function, and numerical bounds, all of which contribute to the stability, robustness, and accuracy of the predicted outcomes. The choice and tuning of genetic operators are critical to the success of training the model to recognize the nonlinear relationships inherent in the experimental data. Figure 8 presents both the predicted versus actual values and the bootstrap distributions of the mean predicted Fp for the training and testing datasets using the GEP model. A strong correlation is observed between the predicted and actual values, as shown in Fig. 8a and c, with R^2^ values of 0.926 and 0.945 for the training and testing datasets, respectively. The accompanying frequency histogram illustrating the distribution and close agreement between predicted and actual values illustrates the model’s robustness in predicting the Fp in SFRCC. To evaluate prediction uncertainty, Fig. 8b and d present the bootstrap distributions of the mean predicted Fp for the training and testing datasets. The 2.5th and 97.5th percentiles define the 95% confidence interval, which shows the range of variation in the model’s predictions. Figure 8 reveals that the GEP proposed model has stable and reliable performance on both the training and testing datasets, thus indicating the robustness and generalizability. Further validation of the model’s accuracy is depicted through the residual analys is presented in Fig. 9. The residual values exhibit a random distribution around the zero reference line, demonstrating homoscedasticity and confirming the absence of systematic trends, biases, or heteroscedastic behavior. The majority of residuals fall within a narrow band, indicating low variability in prediction errors across the predicted Fp. Consequently, this confirms that the proposed model adequately and consistently effectively captures the essential relationships influencing Fp, thus supporting its robustness and predictive reliability. Also, Fig. 10 displays the ratio of actual to predicted Fp, with most data points clustering closely around unity. There are no substantial systematic deviations or biases around the baseline; this distribution supports the high predictive accuracy and stability of the proposed GEP model, which is capable of reliably predicting SFRCC across a wide range of data sets.

**Table 4:** Optimized genetic parameters of the proposed GEP model for predicting fp of SFRCC.

No.	variables	Tuned variables of Fp prediction	No.	variables	Tuned variables of Fp prediction
	General			Genetic operators	
1	Training record	349	11	Mutation value / RNC mutation	0.00206
2	Testing record	88	12	Inversion value	0.00546
3	Number of chromosomes	100	13	Conservative permutation	0.00546
4	Head size	20	14	IS transportation value	0.00546
5	Number of genes	10	15	RIS transportation	0.00546
6	Linking function	Addition	16	One-point recommendation value	0.00277
7	Numerical constants per gene	5	17	Two-point recommendation value	0.00277
8	Data type	Floating-point	18	Gene recombination value	0.00277
9	Lower bound	-100	19	Random closing	0.00102
10	Upper bound	+ 100	20	Random chromosomes	0.00260

### Analysis of the evaluation metrics

**Fig. 11:**
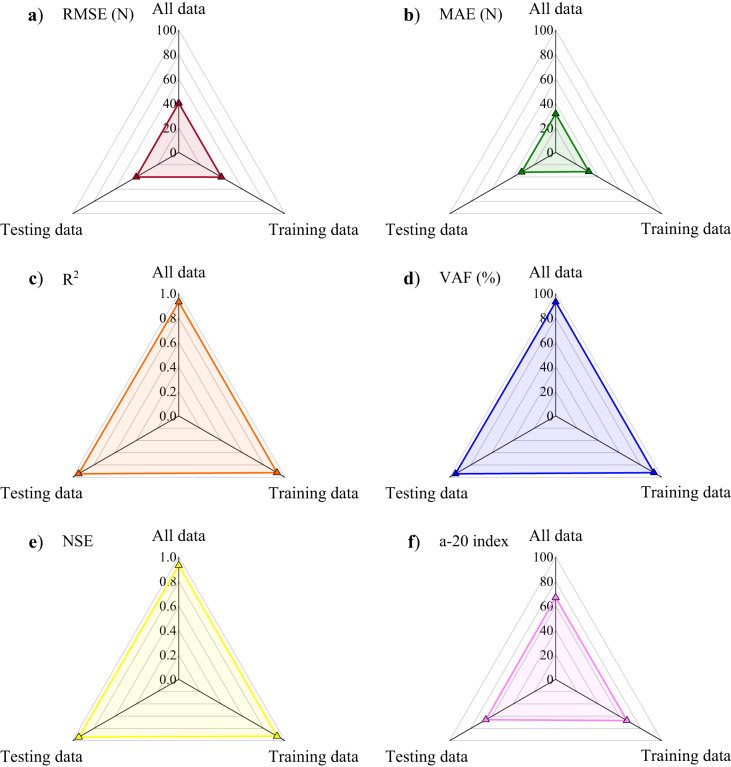
Performance evaluation of the proposed GEP model using various metrics across training, testing, and overall datasets.

The predictive performance of the proposed GEP is assessed using the evaluation metrics, RMSE, MAE, R^2^, VAF, NSE, and the a-20 index, as shown in parts (a) to (f) of Fig. 11, respectively. The RMSE and MAE values of (40.31, 40.10, 40.27) and (31.41, 32.18, 31.57) for the training, testing, and overall datasets, respectively, illustrate consistent and low prediction errors and also confirm the accuracy of the model based on the minimal deviations from observed values. In terms of R^2^, there is a significant correlation between predicted and actual results, with values of (0.926, 0.945, 0.93) for training, testing, and overall datasets, thus confirming the high predictive accuracy of the GEP model. The high VAF values of 92.61%, 94.52%, and 93.01% for the same datasets indicate that the model’s predictions are reliable and consistent. Furthermore, the NSE values of (0.926, 0.942, 0.93) for the same dataset confirm the efficiency of the GEP model in accurately capturing variations in the dataset. Finally, the a-20 index shows that approximately 67% of predictions lie within the acceptable range, specifically 67.04% (training), 65.9% (testing), and 66.81% (overall), underscoring the practical applicability and robustness of the proposed GEP model.

### Analysing k-fold cross-validation

**Fig. 12:**
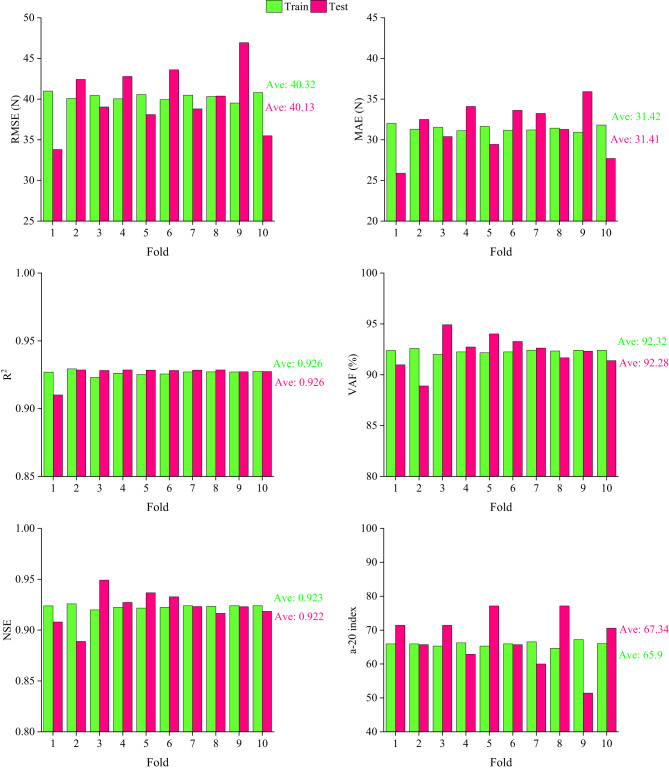
A 10-fold cross-validation results.

To validate the GEP model, a 10-fold cross-validation technique was conducted, as illustrated in Fig. 12. The RMSE range is approximately 35 to 50 N, while the MAE values vary between about 26 and 38 N, which indicates low and consistent prediction errors. Also, the values of R^2^, VAF, and NSE are consistently in the range of 0.90–0.95, while the average a-20 index across all validation folds is above 65%, which indicates the robustness and predictive reliability of the proposed GEP model. The minimal fluctuations observed in these indicators between the training and testing phases across all validation subsets confirm that the proposed model is consistent and generalizable. Consequently, the proposed GEP model maintains consistently accurate and reliable performance, irrespective of the specific dataset used.

### Extra validation of the proposed model

Table 5 shows the extra validation metric to emphasize the precision of the developed model. These include R, adjusted correlation factors (k and k’), and indices of error and stability (R_m_, m, n). The values obtained for R, k, k’, R_m_, m, and n of (0.964, 0.985, 0.992, 0.701, 0.065, 0.071), respectively, all fall within the recommended ranges, confirming that the proposed GEP model is robust, precise, and consistent.

**Table 5:** Validation of the proposed GEP model with extra metrics.

Equation	Recommended range	GEP model
R	> 0.8^86^	0.964
$K=\frac{{\sum\nolimits_{{i=1}}^{n} {({A_i} \times {P_i})} }}{{\sum\nolimits_{{i=1}}^{n} {A_{i}^{2}} }}$	0.85 < k < 1.15^87^	0.985
${K^\prime }=\frac{{\sum\nolimits_{{i=1}}^{n} {({A_i} \times {P_i})} }}{{\sum\nolimits_{{i=1}}^{n} {P_{i}^{2}} }}$	0.85 < k’ < 1.15^87^	0.992
${R_m}={R^2} \times \left( {1 - \sqrt {\left| {{R^2} - R{o^2}} \right|} } \right){\text{ }}$	> 0.5^88^	0.701
$m=\frac{{{R^2} - R{o^2}}}{{{R^2}}}$	< 0.1	0.065
$n=\frac{{{R^2} - Ro{\prime ^2}}}{{{R^2}}}$	< 0.1	0.071
$R{o^2}=1 - \frac{{\sum\nolimits_{{i=1}}^{n} {{{\left( {{P_i} - A_{i}^{o}} \right)}^2}} }}{{\sum\nolimits_{{i=1}}^{n} {{{\left( {{P_i} - P_{i}^{o}} \right)}^2}} }},$ $Ro{\prime ^2}=1 - \frac{{\sum\nolimits_{{i=1}}^{n} {{{\left( {{A_i} - P_{i}^{o}} \right)}^2}} }}{{{{\sum\nolimits_{{i=1}}^{n} {\left( {{A_i} - A_{i}^{o}} \right)} }^2}}}$ ${\text{ A}}_{i}^{o}=k \times {P_i}{\text{ }},$ ${\text{P}}_{i}^{o}={k^\prime } \times {A_i}{\text{ }}$	$\:\cong\:$ 1^88^	

### Comparison of the proposed model with previous studies’ models

To evaluate the performance of the proposed GEP model in this study, a comparative analysis was conducted with two recent studies that utilized various predictive models, including artificial neural networks (ANN), decision trees (DT), and kernel ridge regression (KRR). The evaluation metrics (R^2^, MAE, and RMSE) achieved by the proposed GEP model and those reported in the literature are presented in Table 6. A comparison of the values in the table demonstrates that the GEP model developed in this study outperforms existing models in terms of predictive accuracy. For instance, the R^2^ value obtained here is 0.93, whereas the highest R^2^ values reported for ANN in other studies are 0.91 and 0.90. Additionally, the RMSE value for the GEP model (40.27 N) is approximately 50% lower than the lowest RMSE achieved by other AI-based methods. It should also be noted that the ANN model proposed by Hemmatian et al.^60^ (which achieved an R² of 0.91) was trained on a dataset of 398 samples, a smaller dataset compared to the one used in this study. Consequently, the results presented here are statistically more robust. Furthermore, this study provides a highly accurate and practical GEP-derived formulation for future applications. In contrast, Huang et al.^61^ proposed a formulation with an accuracy of approximately 80%, which exhibits a 13% higher error than the GEP-based formulation developed in this work. These comparative findings validate the superior robustness and reliability of the proposed GEP model over other AI techniques in predicting Fp.

**Table 6:** Performance comparison of the proposed model in this study with previous studies.

Authors (Year) [Ref.]	Proposed model	Evaluation metrics		
		*R* ^2^	MAE (*N*)	RMSE (*N*)
Current study	GEP	0.93	31.57	40.27
Hemmatian et al. (2023)^60^	ANN	0.91	-	-
Huang et al. (2025)^61^	Decision tree	0.89	45.68	97.22
	Kernel ridge regression	0.88	64.38	101.58
	ANN	0.90	46.22	82.81

### Proposed formulation for predicting fp

It is essential for understanding the performance of SFRCC to accurately predict the Fp of fibers in cementitious composites. Therefore, a GEP model was developed to provide a precise formulation for estimating Fp based on key material and geometric factors. Unlike traditional models that use simplified assumptions, this equation accurately represents complex relationships between fiber, concrete, and geometric properties. Also, integrating non-linear interactions better reflects the actual behavior of fibers within the cementitious composite matrix. The predictive model, as shown in Eq. (8), includes multiple input variables that influence the Fp of SFRCC. These variables, labeled as d0 to d7, are defined in Table 7 and selected based on their established relevance and sensitivity in fiber pull-out phenomena.

The most significant advantage of this formulation is its ability to predict Fp without an extensive experimental procedure. It also saves time and resources and helps engineers optimize SFRCC for various applications more effectively. Overall, it provides a practical tool for improving the design and performance of FRCC, making structures more durable and efficient.

8 $$\begin{aligned} Fp & = \left[ {d_{3}^{3} } \right] + \left[ {d_{3} \times \left( {d_{4} - \frac{{123.13d_{3} (2d_{6}^{2} + d_{6} - (d_{2}^{{0.5}} (92.81d_{6} ))}}{{d_{2}^{2} }}} \right)} \right] \\ & \;\;\; + \left[ {d_{4} - d_{7} - \frac{{d_{1} }}{{\left( {d_{3} \times (2d_{5} - 86 - \frac{{d_{2} }}{{d_{5} }} + d_{7} )^{4} } \right)^{{0.166}} }}} \right] + \left[ {d_{1} d_{4}^{{0.5}} d_{7} } \right] \\ & \;\;\; + \left[ {\frac{{ - 15.25(d_{5} - 15.25)^{5} }}{{\left( {\frac{{546.795 \times 10^{3} }}{{d_{4} d_{7} - 63.62}}} \right)^{3} - d_{6}^{4} }}} \right] + \left[ {\frac{{d_{4}^{3} }}{{d_{7} \times \left( {\frac{{d_{5}^{2} }}{{d_{2} }} + d_{4}^{2} \times d_{0} \times d_{7}^{5} \times \frac{{d_{0}^{2} }}{{1653.23}}} \right)}}} \right]^{{0.5}} \\ & \;\;\; + \left[ {\frac{{(70.23 - d_{7}^{{0.5}} )^{6} }}{{d_{2}^{3} }}} \right] + \left[ {\left( {\frac{{d_{3} d_{2} \times 79.7 \times 10^{5} }}{{d_{5}^{5} \left( {\frac{{d_{2} + d_{5} }}{{ - 75.33 + d_{4} }} + 81.14} \right)}}} \right)^{2} + 94.49 + d_{4} } \right] \\ & \;\;\; + \left[ {d_{3} - \sqrt {1861.05 + d_{6} } - 77.90 - d_{5} - d_{7} } \right] + \left[ {d_{7} d_{1} d_{4}^{{0.5}} } \right] \\ \end{aligned}$$

**Table 7:** Definition of input variable in the proposed formula.

	Input variables							
	α	f’c	ft	Geometry	Le	Lf/df	L.rate	w/c
Label	d0	d1	d2	d3	d4	d5	d6	d7

Equation (9) presents the full mathematical expression of Eq. (8), including a complete symbolic representation of all input variables.

9 $$\begin{aligned} Fp & = \left[ {Geometry^{3} } \right] + \left[ {Geometry \times \left( {L_{e} - \frac{{123.13Geometry(2L.rate^{2} + L.rate - (ft^{{0.5}} (92.81L.rate))}}{{ft^{2} }}} \right)} \right] \\ & \;\;\; + \left[ {L_{e} - (w/c) - \frac{{f^{\prime}c}}{{(Geometry \times (2(Lf/df) - 86 - \frac{{ft}}{{(Lf/df)}} + (w/c))^{4} )^{{0.166}} }}} \right] + \left[ {f^{\prime}c \times L_{e}^{{0.5}} (w/c)} \right] \\ & \;\;\; + \left[ {\frac{{ - 15.25((Lf/df) - 15.25)^{5} }}{{\left( {\frac{{546.795 \times 10^{3} }}{{L_{e} (w/c) - 63.62}}} \right)^{3} - L.rate^{4} }}} \right] + \left[ {\frac{{L_{e}^{3} }}{{(w/c) \times \left( {\frac{{(Lf/df)^{2} }}{{ft}} + L_{e}^{2} \times \alpha \times (w/c)^{5} \times \frac{{\alpha ^{2} }}{{1653.23}}} \right)}}} \right]^{{0.5}} \\ & \;\;\; + \left[ {\frac{{(70.23 - (w/c)^{{0.5}} )^{6} }}{{ft^{3} }}} \right] + \left[ {\left( {\frac{{Geometry \times ft \times 79.7 \times 10^{5} }}{{(Lf/df)^{5} \left( {\frac{{ft + (Lf/df)}}{{ - 75.33 + L_{e} }} + 81.14} \right)}}} \right)^{2} + 94.49 + L_{e} } \right] \\ & \;\;\; + \left[ {Geometry - \sqrt {1861.05 + L.rate} - 77.90 - (Lf/df) - (w/c)} \right] + \left[ {(w/c) \times f^{'} c \times L_{e}^{{0.5}} } \right] \\ \end{aligned}$$

### Influence of input variables on model’s prediction by SHAP

**Fig. 13:**
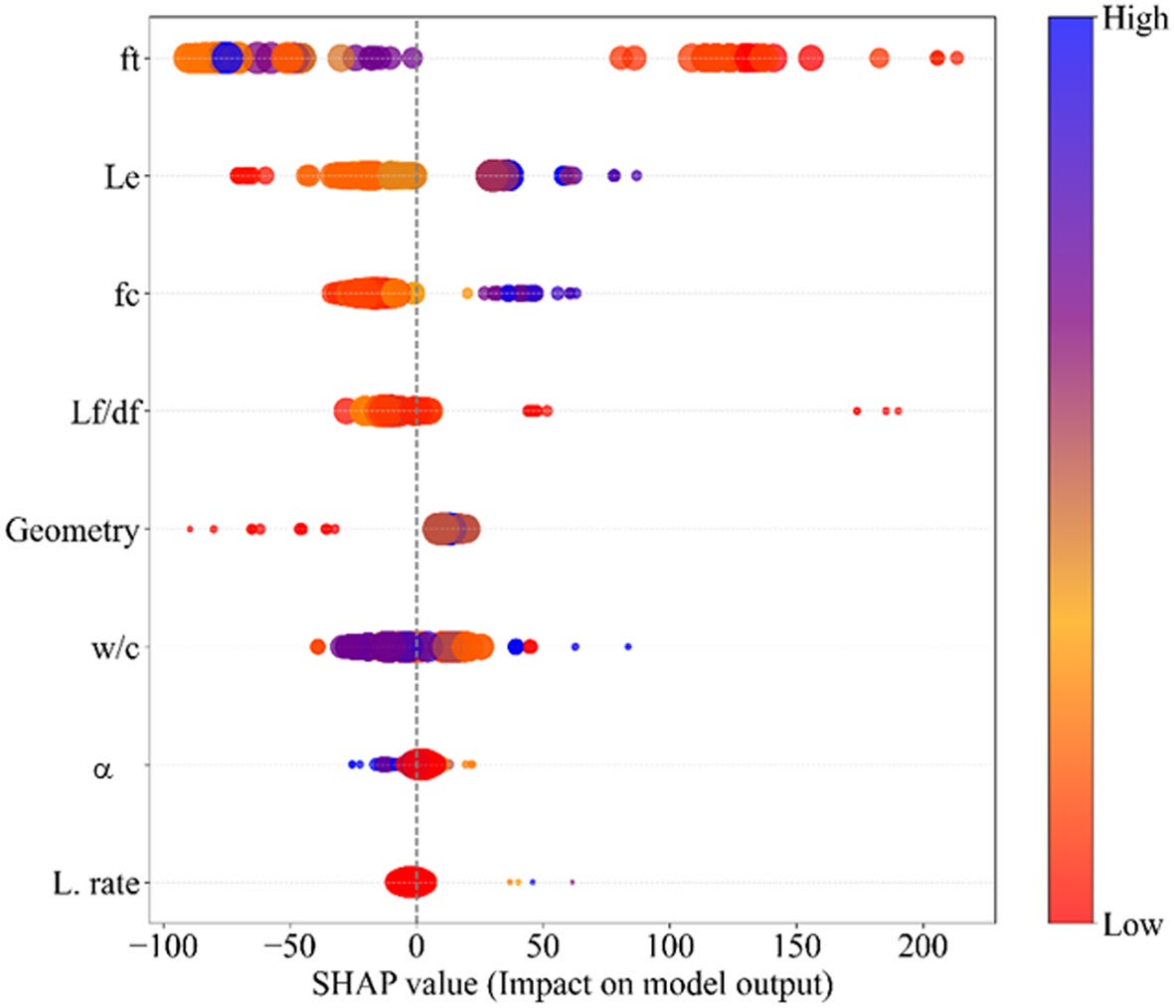
SHAP analysis for SFRCC illustrating the positive and negative influence of input variables on the Fp.

**Fig. 14:**
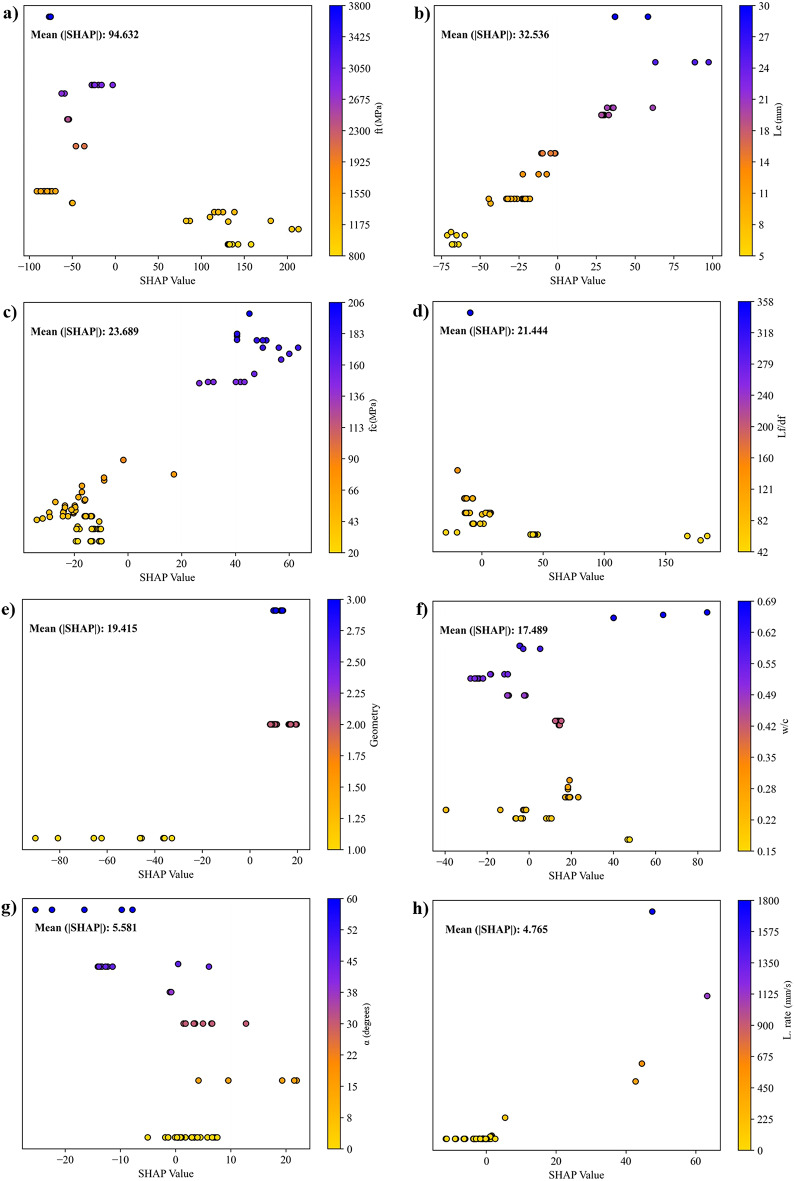
Summary plots of SHAP analysis in SFRCC regarding the effects of variables: (a) ft, (b) L_e_, (c) f’c, (d) Lf/df, (e) Geometry, (f) w/c, (g) α, and (h) L. rate.

To better interpret the GEP model’s prediction behavior and the importance of the input variables, the SHAP analysis was applied and visualized in Figs. 13 and 14. These illustrations show how the input variables affect the model’s output. The color gradient ranges from blue (high feature values) to red (low feature values), which provides further insight into how each variable impacts. It can be derived from Figs. 13 and 14 that among all variables, ft is the most influential variable, with SHAP values ranging from − 100 to + 200 and a mean absolute value of 92.385. However, it is shown that higher ft values lead to lower predicted results, while lower values improve it. Similarly, L_e_ also plays a notable role, exhibiting SHAP values between − 50 and + 100, indicating a more balanced but still meaningful effect. Thus, SHAP results in Fig. 14a and b indicate that ft negatively influences Fp, whereas L_e_ positively contributes, which illustrates their contrasting roles in fiber–matrix interaction. Besides, f’c shows a notable effect, with SHAP values rising to + 60, which indicates its essential role in structural performance. However, the strong positive contribution indicates that higher compressive strength values can significantly improve the predicted results, while lower values suppress it. Another key variable is Lf/df, with SHAP values reaching + 150, highlighting the importance of this variable in shaping predictions. The model assigns lower values of Lf/df a positive impact, suggesting that decreasing the aspect ratio enhances the predicted result. Furthermore, geometry has a moderate influence, with most SHAP values within ± 25, suggesting that while shape-related factors matter, their impact is less noticeable than strength-related variables. The w/c shows an inverse relationship, where lower values tend to improve predictions, aligning with material science principles. Meanwhile, the α and L.rate contribute minimally, with SHAP values mostly within ± 20, indicating a secondary influence on the model’s predictions.

Overall, the SHAP analysis confirms that mechanical properties, particularly tensile and compressive strength, are the primary influential factors in the model’s predictions, while geometric and process-related factors serve as secondary influences. These findings enhance model interpretability and support future material optimization and are in accordance with previous findings reported in^89,90^.

## Conclusions

This study developed a data-driven approach utilizing GEP to predict the pull-out force of steel fibers in SFRCC. The model was trained and tested on a comprehensive dataset using carefully optimized genetic parameters, which led to accurate capture of the complex nonlinear relationships between fiber properties, matrix characteristics, and fiber geometric factors. Also, it has been evaluated with statistical criteria for a superior validation. Therefore, the key findings of this study are highlighted below:

- The GEP model demonstrated high accuracy by achieving an R^2^ value of 0.93, which indicates robust agreement between predicted and actual Pull-out force values.
- The proposed GEP prediction model presented an acceptable predicting ability, which is revealed by statistical evaluation measures, including a VAF = 93.01%, RMSE = 40.27 and NSE of 0.93.
- K-fold cross-validation confirmed the model’s strength and capability to generalize well to unseen data with consistent performance and minimal fluctuations in error metrics among all folds.
- Unlike traditional AI models, the GEP model proposed an interpretable equation for Fp prediction, which is practical for engineering and further research. This formulation integrates key material and geometric variables, offering a practical tool to optimize SFRCC design without extensive experimentation.
- Sensitivity analysis using SHAP interpretation determined fiber tensile strength and embedment length as the most influential factors impacting the Pull-out force, with ft showing a pronounced negative impact on predictions. Compressive strength (f’c) and aspect ratio (Lf/df) also played significant roles, while α, L.rate, etc. had secondary effects.

## Limitations and future directions

While the proposed GEP model provides a reliable approach for predicting steel fiber pull-out force, it could be further studied in future work by investigating additional fiber characteristics like surface roughness and coating materials, as well as other fiber types beyond steel. The model’s accuracy might be further improved using hybrid AI techniques. Future validation with larger datasets, including diverse mix designs, loading conditions, and microstructural parameters obtained through advanced imaging techniques, would enhance generalizability. Additionally, exploring its use in sustainable material development, such as recycled fiber composites, could expand its environmental relevance while maintaining predictive capability.

## Supplementary Information

Below is the link to the electronic supplementary material.

Supplementary Material 1

## Data Availability

The database used in this study is available in the supplementary file.
